# MMP-2 is a novel histone H3 N-terminal protease necessary for myogenic gene activation

**DOI:** 10.1186/s13072-021-00398-4

**Published:** 2021-05-17

**Authors:** Judd C. Rice, Benjamin H. Weekley, Tomas Kanholm, Zhihui Chen, Sunyoung Lee, Daniel J. Fernandez, Rachel Abrahamson, Alessandra Castaldi, Zea Borok, Brian D. Dynlacht, Woojin An

**Affiliations:** 1 Department of Biochemistry and Molecular Medicine, Norris Comprehensive Cancer Center, University of Southern California Keck School of Medicine, 1450 Biggy Street, HNRT 6506, Los Angeles, CA 90033 USA; 2 Department of Molecular Microbiology and Immunology, Norris Comprehensive Cancer Center, University of Southern California Keck School of Medicine, Los Angeles, CA 90033 USA; 3 Hastings Center for Pulmonary Research and Division of Pulmonary, Critical Care and Sleep Medicine, Department of Medicine, Keck School of Medicine, University of Southern California, Los Angeles, CA 90033 USA; 4 Department of Pathology and Cancer Institute, NYU School of Medicine,, New York, NY 10016 USA

## Abstract

**Background:**

Selective proteolysis of the histone H3 N-terminal tail (H3NT) is frequently observed during eukaryotic development, generating a cleaved histone H3 (H3cl) product within a small, but significant, portion of the genome. Although increasing evidence supports a regulatory role for H3NT proteolysis in gene activation, the nuclear H3NT proteases and the biological significance of H3NT proteolysis remain largely unknown.

**Results:**

In this study, established cell models of skeletal myogenesis were leveraged to investigate H3NT proteolysis. These cells displayed a rapid and progressive accumulation of a single H3cl product within chromatin during myoblast differentiation. Using conventional approaches, we discovered that the canonical extracellular matrix (ECM) protease, matrix metalloproteinase 2 (MMP-2), is the principal H3NT protease of myoblast differentiation that cleaves H3 between K18-Q19. Gelatin zymography demonstrated progressive increases in nuclear MMP-2 activity, concomitant with H3cl accumulation, during myoblast differentiation. RNAi-mediated depletion of MMP-2 impaired H3NT proteolysis and resulted in defective myogenic gene activation and myoblast differentiation. Supplementation of MMP-2 ECM activity in MMP-2-depleted cells was insufficient to rescue defective H3NT proteolysis and myogenic gene activation.

**Conclusions:**

This study revealed that MMP-2 is a novel H3NT protease and the principal H3NT protease of myoblast differentiation. The results indicate that myogenic signaling induces MMP-2-dependent H3NT proteolysis at early stages of myoblast differentiation. Importantly, the results support the necessity of nuclear MMP-2 H3NT protease activity, independent of MMP-2 activity in the ECM, for myogenic gene activation and proficient myoblast differentiation.

## Introduction

Developmental signaling cascades ultimately converge in the nucleus to induce broad, yet precise, epigenomic changes that facilitate the activation of lineage-specific factors necessary for differentiation. One of the epigenomic changes frequently observed during differentiation is the limited and selective proteolysis of the histone H3 N-terminal tail (H3NT) that generates a cleaved histone H3 (H3cl) product within a significant portion of the genome [1–4]. Previous reports demonstrated that an H3cl product is frequently observed within chromatin of various animal tissues and a diverse array of eukaryotes ranging from single cell organisms, including *S. cerevisiae* and *T. thermophila*, to multicellular animals such as *D. melanogaster*, *M. musculus* and *H. sapiens* [5–10]. More recent studies indicate that H3NT proteolysis is a consistent feature of eukaryotic developmental programs including yeast sporulation, mouse spermatogenesis, mouse and human embryonic stem cell differentiation, and differentiation of specialized tissues including breast, bone and skeletal muscle [11–15]. Although these collective studies support H3NT proteolysis as an evolutionarily conserved event in development, the functional and biological significance of this epigenetic modification remain largely unknown.

One obvious functional role of H3NT proteolysis is the “erasure” of existing H3NT post-translational modifications (PTMs) and their associated binding proteins/complexes that likely expedites robust changes in gene expression observed during differentiation. This mechanism is consistent with observations reported in yeast where H3NT proteolysis “erased” repressive H3NT PTMs and resulted in gene activation [15]. Another likely function of H3NT proteolysis is nucleosome and chromatin remodeling. Early structural studies demonstrated that the histone NTs are necessary for the stable assembly of nucleosomes and competent formation of a higher order solenoid chromatin structure [16–18]. More recent reports confirmed that lack of an H3NT destabilizes intra- and inter-nucleosomal interactions, both in *cis* and *trans* [19–23]. While these in vitro studies support a function for H3NT proteolysis in ATP-independent chromatin remodeling that increases accessibility to DNA-binding factors, such as transcription factors, this has yet to be experimentally validated in cells.

Despite its discovery over 55 years ago, important insights into the biological significance of H3NT proteolysis have only occurred recently due, in large part, to the identification of several H3NT proteases [24, 25]. The first H3NT protease identified, Cathepsin L, is a canonical lysosomal protease that functions as a nuclear H3NT protease during mouse embryonic stem cell differentiation and senescence of IMR90 cells [8, 26]. Another lysosomal protease, Cathepsin D, facilitates H3NT proteolysis during mouse mammary development [13]. We recently discovered that matrix metalloproteinase 9 (MMP-9) functions as a nuclear H3NT protease during osteoclastogenesis [14]. Although MMP-9 is a canonical extracellular matrix (ECM) protease, we observed the rapid and progressive accumulation of nuclear MMP-9 activity, concomitant with H3NT proteolysis, following RANKL-induced differentiation of osteoclast precursor cells. We also found that MMP-9-dependent H3NT proteolysis was required for activation of osteoclastogenic genes and proficient osteoclast differentiation. These collective studies support an innovative model where distinct non-nuclear proteases are utilized as nuclear H3NT protease, in a developmental-dependent context, to facilitate the activation of appropriate lineage-specifying genes necessary for proficient differentiation.

Based on this model, we hypothesized that a novel H3NT protease was responsible for H3NT proteolysis observed in the established mouse C2C12 model of myoblast differentiation [11]. As predicted, we discovered that matrix metalloproteinase 2 (MMP-2) is the principal H3NT protease of myoblast differentiation. Consistent with our previous findings, we observed the rapid and progressive accumulation of nuclear MMP-2 activity, concomitant with H3NT proteolysis, during myoblast differentiation. Our results demonstrate that MMP-2-dependent H3NT proteolysis is required for myogenic gene activation and proficient myoblast differentiation. Importantly, this study provides the first experimental evidence to support the necessity of nuclear H3NT protease activity, independent of its canonical non-nuclear activity, for gene activation.

## Results

### Myoblast differentiation induces N-terminal proteolysis of chromatin-bound histone H3

**Fig. 1:**
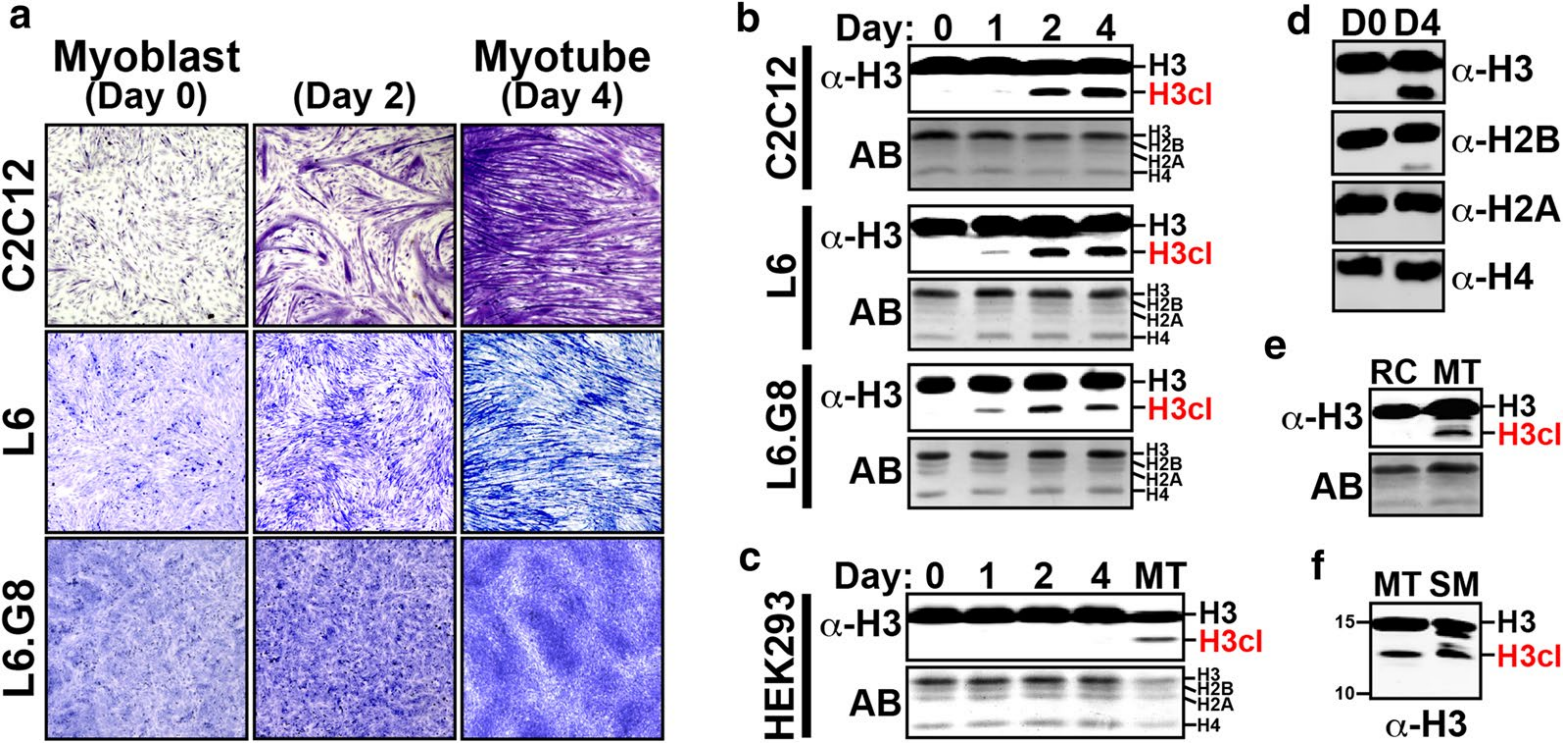
Accumulation of a chromatin-bound H3cl product during skeletal myogenesis. **a** Light microscopy (10x) of LADD stained mouse C2C12 and rat L6 myoblasts (day 0) cultured in differentiation media that induces cell fusion, multinucleation and formation of striated myotubes (day 4). The L6.G8 myoblasts, a derivative of L6, do not form myotubes. **b** A histone H3 C-terminal antibody was used for Western analysis of chromatin purified from myoblasts and human HEK293 kidney cells **c** cultured in differentiation media for the indicated number of days. Proteolysis of the H3 N-terminus (H3NT) generates a fast-migrating cleaved H3 (H3cl) product as indicated. Amido black staining (AB) of membranes was performed to confirm equivalent loading of chromatin between samples. **d** Western analysis of chromatin isolated from C2C12 myoblasts (D0) and myotubes (D4) using antibodies to detect proteolysis of core histones H3, H2B, H2A or H4. **e** Western analysis of chromatin of C2C12 myotubes (MT) enriched from the remaining undifferentiated reserve cells (RC) after 4 days in differentiation media. **f** Representative Western analysis of chromatin extracted from adult mouse skeletal muscle (SM) tissue compared to C2C12 myotube (MT) chromatin. The protein ladder indicates the relative size of histones H3 (15 kDa) and H4 (10 kDa)

To investigate the timing of H3NT proteolysis during myoblast differentiation, mouse C2C12 cells were expanded to confluence in growth media before replacement with differentiation media to induce synchronous myoblast differentiation [11]. Within 2 days following media replacement, the small mononuclear myoblasts begin to fuse and ultimately form long striated multinuclear myotubes by Day 4 (Fig. 1a). Chromatin purified from C2C12 cells at defined time points during differentiation was processed for Western analysis using an antibody that detects the C-terminus of histone H3. Compared to the myoblast precursor cells, chromatin of differentiating myoblasts displayed the appearance of a fast-migrating H3 band between 24–48 h following media replacement that increased during myoblast differentiation (Fig. 1b). The fast-migrating H3 band is indicative of a chromatin-bound cleaved H3 (H3cl) product generated by proteolysis of the histone H3 N-terminus (H3NT) [14]. To corroborate these findings, the experiments were repeated using the rat L6 myoblast cell line, which undergo myoblast differentiation analogous to mouse C2C12 cells (Fig. 1a). The results were strikingly similar to C2C12 cells, demonstrating that H3NT proteolysis was induced within 24 h following media replacement resulting in the progressive accumulation of a single H3cl product in differentiating L6 myoblast and myotube chromatin (Fig. 1b). In contrast, chromatin from human HEK293 kidney cells cultured in differentiation media lacks an H3cl product supporting H3NT proteolysis as a defining feature of myoblast differentiation rather than an experimental artifact of media replacement (Fig. 1c). Western analysis also demonstrated that chromatin-bound histone H3 is the principal core histone targeted for proteolysis during myoblast differentiation (Fig. 1d). Furthermore, the H3cl product was specifically enriched in chromatin of purified C2C12 myotubes but absent in chromatin of the residual non-differentiated mononuclear reserve cells isolated from the same culture (Fig. 1e) [27]. These results further support the induction of H3NT proteolysis as a programmed epigenetic event of myoblast differentiation.

### Cleaved H3 is present in skeletal muscle chromatin

The accumulation and retention of an H3cl product in C2C12 and L6 myotube chromatin predicted that an H3cl product would also be present in mouse skeletal myofibers. To test this, thigh muscle from adult mouse hind limb was dissected and chromatin from purified muscle tissue was processed for Western analysis [28]. The results confirmed the presence of a prominent H3cl product in muscle tissue chromatin with similar molecular size as observed in C2C12 and L6 myotube chromatin (Fig. 1f). These collective findings support H3NT proteolysis as a conserved feature of skeletal myogenesis in vitro and in vivo and that the resulting H3cl product is, at least partially, retained in skeletal myofibers.

### Myogenic signaling induces H3NT proteolysis prior to myoblast fusion

**Fig. 2:**
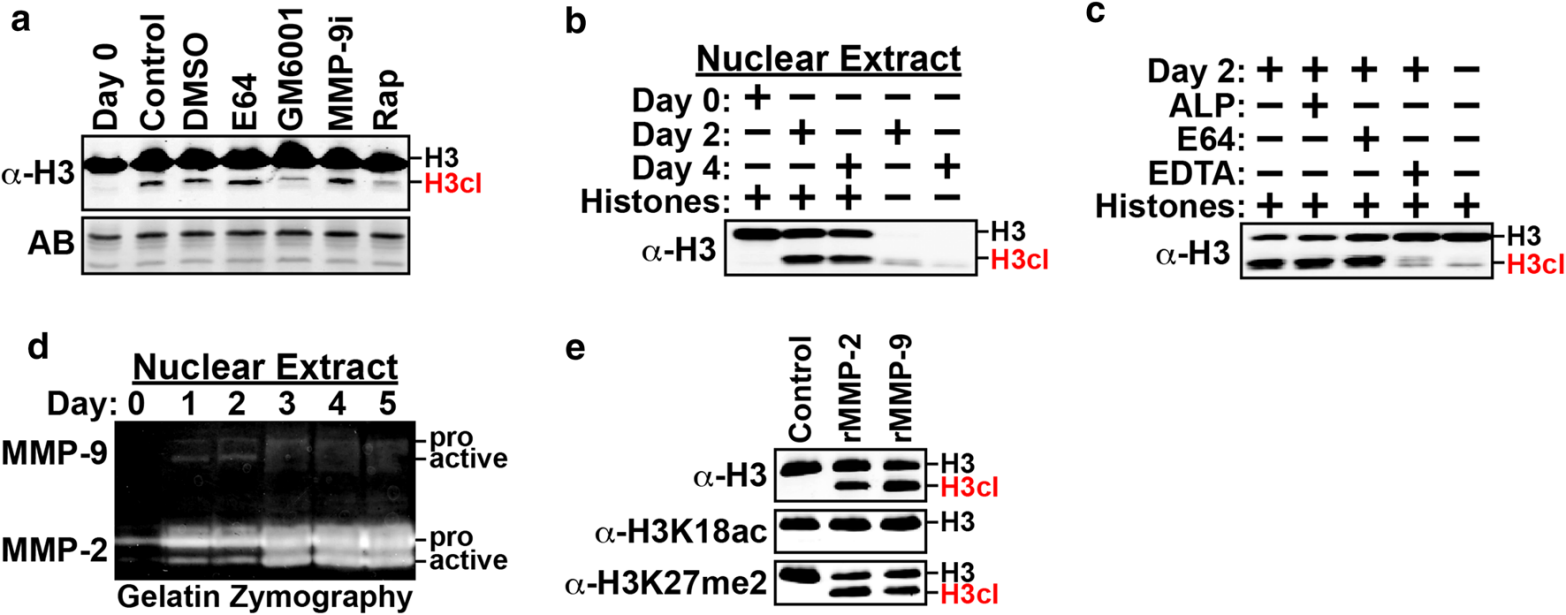
MMP-2 is a novel H3NT protease that localizes to the nucleus during myoblast differentiation. **a** Western analysis to detect the H3cl product in chromatin of C2C12 cells before (Day 0) and after culturing in differentiation media (Day 4) supplemented with DMSO (vehicle) or the inhibitors E64 (cathepsin), GM6001 (MMP), MMP-9i (MMP-9 specific) or rapamycin (myogenic). Amido black staining (AB) of membranes was performed to confirm equivalent loading of chromatin between samples. **b** In vitro H3 cleavage assay using nuclear soluble extracts purified from C2C12 myoblasts (day 0), differentiating cells (day 2) and myotubes (day 4) incubated alone or with HeLa core histone substrates. Western analysis was performed to detect a cleaved H3 (H3cl) product. (**c**) In vitro H3 cleavage assay using nuclear soluble extracts from differentiating C2C12 cells (day 2) incubated with selective inhibitors of specific protease families prior to addition of core histone substrates. Inhibitors include a cocktail of aprotinin (serine), leupeptin (serine/cysteine) and pepstatin (aspartyl), E64 (cathepsin) or EDTA (MMP). **d** Representative gelatin zymography using equivalent amounts of nuclear soluble extracts collected from differentiating C2C12 cells at the indicated time points. The proform of MMP-9 (92 kDa) and MMP-2 (72 kDa) and their cleaved active forms are indicated. **e** In vitro H3 cleavage assay using core histone substrates in the presence or absence of active recombinant MMP-2 or MMP-9. Western analysis was performed to detect the H3cl product using the indicated antibodies

The findings above demonstrate that the H3cl product accumulates in C2C12 and L6 chromatin shortly after the onset of differentiation, suggesting that activation of H3NT proteolysis occurs prior to myoblast fusion. However, the absence of an H3cl product in the mononuclear HEK293 and C2C12 reserve cell chromatin alternatively suggests that activation of H3NT proteolysis is dependent on myoblast fusion. To resolve these possibilities, the experiments were repeated using a clonal derivative of L6 cells, L6.G8, that are incapable of myoblast fusion but retain the myogenic signaling mechanisms necessary to initiate the differentiation program (Fig. 1a) [29]. Similar to the parental L6 results, Western analysis demonstrated the appearance of a single H3cl product in L6.G8 chromatin within 24 h after media replacement, indicating that induction of H3NT proteolysis is independent of myoblast fusion (Fig. 1b). To investigate the dependence of H3NT proteolysis on myogenic signaling, C2C12 cells were cultured in differentiation media supplemented with the potent myogenic inhibitor, rapamycin [30]. Western analysis demonstrated that inhibition of myogenic signaling by rapamycin resulted in the depletion of the H3cl product relative to control (Fig. 2a). These findings indicate that myogenic signaling facilitates induction of H3NT proteolysis at earlier stages of the differentiation program, prior to myoblast fusion.

### MMP activity is required for H3NT proteolysis during myoblast differentiation

The data indicate that an unknown protease functions in the nucleus to directly cleave the H3NT within chromatin during myoblast differentiation. To identify this protease, a series of in vitro H3 cleavage assays were first performed by incubating HeLa core histone substrates with nuclear soluble extracts purified from C2C12 myoblasts (day 0), differentiating myoblasts (day 2) or myotubes (day 4) [14]. Western analysis of the samples demonstrated robust H3NT protease activity in nuclear extracts of differentiating myoblasts and myotubes, but not C2C12 myoblasts, resulting in the generation of a single H3cl product (Fig. 2b). These in vitro results, which mirrored the timing of H3NT proteolysis and size of the H3cl product during myoblast differentiation (Fig. 1b), confirmed the presence of a nuclear H3NT protease in differentiating myoblasts. The in vitro H3 cleavage assays were repeated using nuclear soluble extracts preincubated with selective inhibitors of the serine, cysteine, aspartyl, cathepsin or MMP families of proteases. Western analysis demonstrated that only inhibition of MMP activity impaired H3NT protease activity, indicating that the nuclear H3NT protease is an MMP (Fig. 2c). To test the necessity of MMP activity for H3NT proteolysis during myoblast differentiation, C2C12 cells were cultured in differentiation media supplemented with the cell permeable chemicals E64 or GM6001 that broadly inhibit cathepsin or MMP activity, respectively. Western analysis demonstrated that cathepsin inhibition did not impair generation of an H3cl product within chromatin of differentiating myoblasts relative to vehicle and rapamycin controls (Fig. 2a). Conversely, inhibition of MMP activity resulted in a significant reduction of the H3cl product, indicating that MMP activity is required for H3NT proteolysis during myoblast differentiation.

### MMP-2 is a novel H3NT protease

Since MMP-9 is the only MMP previously reported to function as an H3NT protease, we hypothesized that MMP-9 was similarly responsible for H3NT proteolysis during myoblast differentiation [14]. Contrary to the hypothesis, however, selective chemical inhibition of MMP-9 was insufficient to impede H3NT proteolysis in differentiating C2C12 cells (Fig. 2a). To validate these negative results, gelatin zymography was performed to assess nuclear MMP gelatinase activity using nuclear soluble extracts purified from differentiating C2C12 myoblasts. The progressive accumulation of nuclear MMP-9 activity observed during osteoclastogenesis was not observed during myoblast differentiation (Fig. 2d) [14]. Gelatin zymography did, however, reveal the progressive and robust accumulation of nuclear MMP-2 activity in differentiating C2C12 cells (Fig. 2d). MMP-2 and MMP-9 comprise the gelatinase subfamily of MMPs and are structurally similar with overlapping substrate specificities [31]. The gelatinases are synthesized as a latent proenzyme and subsequently converted to an enzymatically active form following proteolysis of their inhibitory N-terminal prodomain. The observed increase in the enzymatically active form of nuclear MMP-2 during differentiation mirrored the increase of H3NT proteolysis (Fig. 1b), suggesting that MMP-2 is the H3NT protease of myoblast differentiation. To test this directly, the in vitro H3 cleavage assay was performed using recombinant MMP-2 and MMP-9 as the positive control. The results confirmed that MMP-2 is an H3NT protease that generates an H3cl product of comparable size to MMP-9 (Fig. 2e). The MMP-9 gelatinase was previously reported to cleave H3 between K18-Q19 suggesting that the MMP-2 gelatinase also cleaves H3 between K18-Q19 [14]. To test this, Western analysis was performed on the samples using different H3 PTM-specific antibodies. Consistent with a previous report, an H3K27me2 antibody detected the H3cl product whereas an H3K18ac antibody did not (Fig. 2e) [14]. These findings support MMP-2 as a novel H3NT protease that cleaves H3 between K18-Q19.

### MMP-2 is the principal H3NT protease of myoblast differentiation

**Fig. 3:**
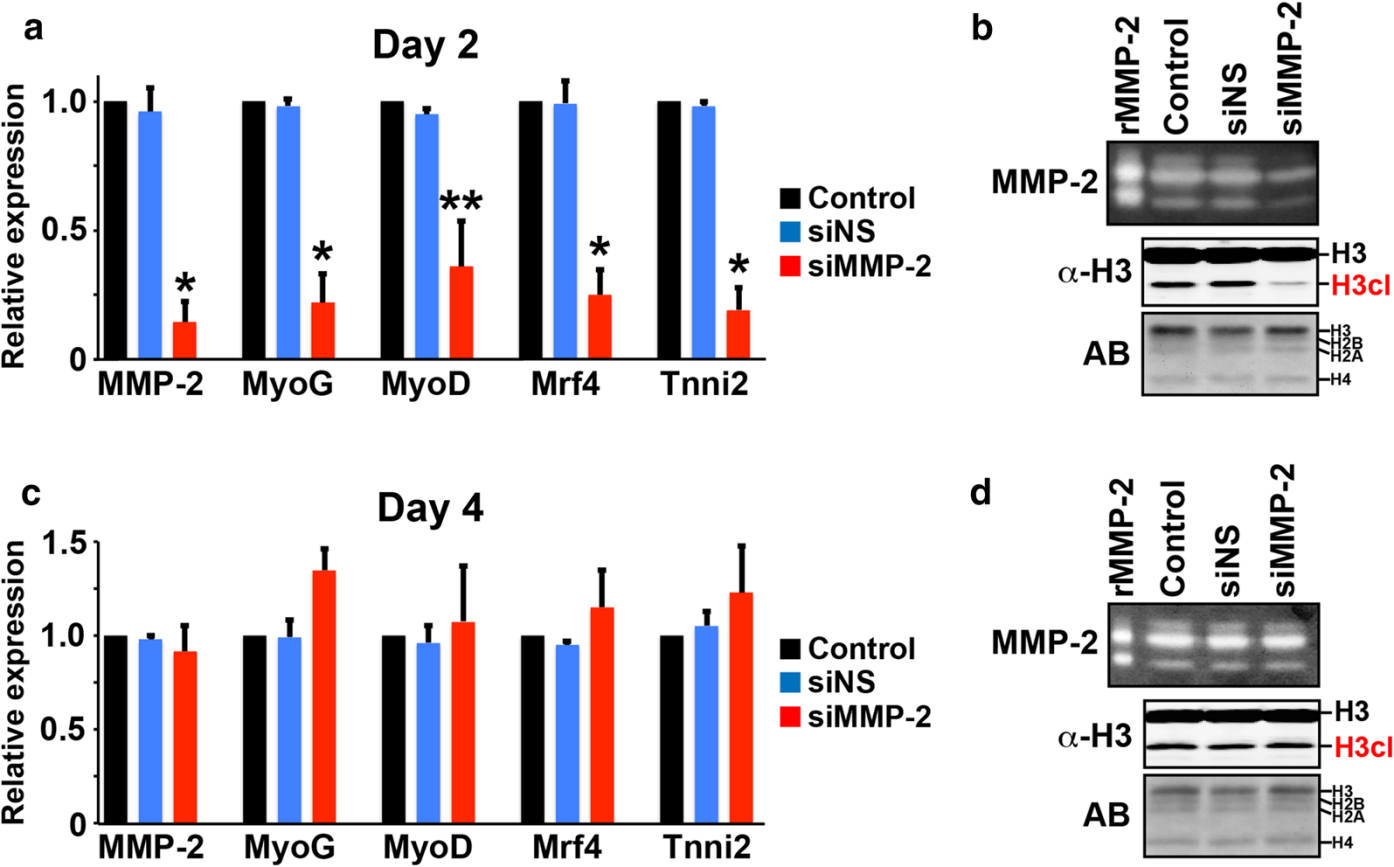
Myogenic gene activation is dependent on MMP-2. **a** RT-qPCR analysis of total RNA purified from control and C2C12 cells transiently transfected with a non-specific siRNA (siNS) or MMP-2-specific siRNA (siMMP-2) and cultured in differentiation media for 2 days. qPCR was performed for MMP-2 and the indicated myogenic genes (x-axis), normalized to the 18S housekeeping gene and plotted relative to C2C12 control for each gene (y-axis). Three independent biological replicates were performed to generate standard deviation (error bars). The Student’s *t* test was used to determine statistical significance between control and siMMP-2: *p* < 0.01 (*) and *p* < 0.03 (**). **b** Gelatin zymography of recombinant MMP-2 and cultured media collected from control, siNS and siMMP-2 day 2 C2C12 cells (top). Western analysis to detect the H3cl product in purified chromatin (middle). Amido black staining (AB) of membranes was performed to confirm equivalent loading of chromatin between samples (bottom). **c** RT-qPCR analysis as described above for control, siNS and siMMP-2 C2C12 cells cultured in differentiation media for 4 days. **d** Gelatin zymography and Western analysis as described above for control, siNS and siMMP-2 C2C12 cells cultured in differentiation media for 4 days

The results above suggested that MMP-2 functions as the H3NT protease during myoblast differentiation. To confirm this, transient transfections were performed in C2C12 cells using an MMP-2-specific siRNA or a non-specific siRNA (NS) as the negative control. Cells were transfected during seeding and expanded to confluence before replacement with differentiation media. Total RNA, cultured media and chromatin were collected for analyses at earlier (day 2) and later (day 4) times after media replacement. RT-qPCR analysis of day 2 cells confirmed an ~ 80% reduction of MMP-2 expression in the siMMP-2 cells relative to the controls (Fig. 3a). Depletion of MMP-2 expression resulted in the concomitant reduction of MMP-2 protease activity, as observed by gelatin zymography using cultured media collected from day 2 cells (Fig. 3b). Consistent with the hypothesis, Western analysis confirmed that reduced MMP-2 activity resulted in impaired H3NT proteolysis in the siMMP-2 day 2 cells relative to the controls (Fig. 3b). Although these results predicted that the siMMP-2 day 4 cells would continue to display defective H3NT proteolysis, the transient effects of siRNA were eliminated in Day 4 cells as demonstrated by restoration of MMP-2 expression and MMP-2 activity to levels similar to those observed in the controls (Fig. 3c, d). Importantly, restoration of MMP-2 activity resulted in the complete rescue of H3NT proteolysis (Fig. 3d). These collective results support MMP-2 as the principal H3NT protease of myoblast differentiation.

### MMP-2 facilitates myogenic gene activation

It was previously reported that MMP-9-mediated H3NT proteolysis facilitates the activation of osteoclastogenic genes necessary for proficient differentiation of osteoclast precursor cells [14]. Based on these findings we hypothesized that MMP-2-mediated H3NT proteolysis, likewise, is necessary to facilitate activation of myogenic genes during myoblast differentiation. To test this hypothesis, RT-qPCR was performed to assess expression changes of the canonical myogenic genes MyoG, MyoD, Mrf4 and Tnni2 in the C2C12 siMMP-2 and control cells [32, 33]. Results from the siMMP-2 day 2 cells confirmed the hypothesis, demonstrating that reduced MMP-2 H3NT protease activity impaired activation of all myogenic genes assayed (Fig. 3a, b). Importantly, restoration of MMP-2 H3NT protease activity in the siMMP-2 day 4 cells resulted in the complete rescue of myogenic gene expression to levels similar to the controls (Fig. 3c, d). These data demonstrate the necessity of MMP-2 for proficient myogenic gene activation during myoblast differentiation.

### MMP-2 is necessary for proficient myoblast differentiation

**Fig. 4:**
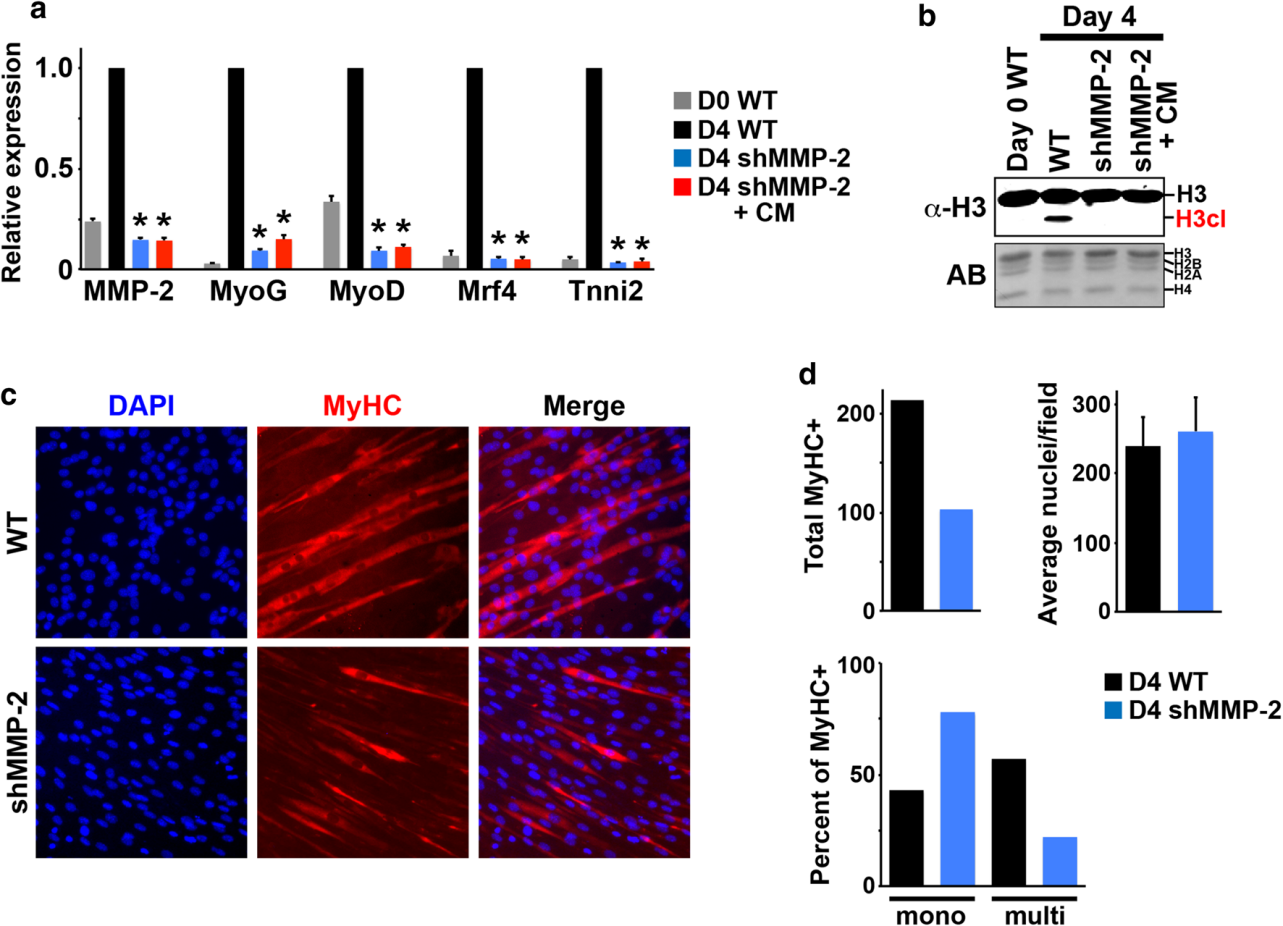
Nuclear MMP-2 activity is required for H3NT proteolysis and myogenic gene activation. **a** RT-qPCR analysis of total RNA purified from C2C12 wild type myoblasts (D0 WT) and C2C12 cells expressing an MMP-2-specific shRNA (shMMP-2) cultured in differentiation media or MMP-2-rich conditioned media (+CM) for 4 days. qPCR was performed for MMP-2 and the indicated myogenic genes (x-axis), normalized to the 18S housekeeping gene and plotted relative to C2C12 day 4 wild type control (D4 WT) for each gene (y-axis). Three independent biological replicates were performed to generate standard deviation (error bars). The Student’s *t* test was used to determine statistical significance between D4 WT control and D4 shMMP-2: *p* < 10^–6^(*). **b** Western analysis to detect the H3cl product in chromatin purified from the C2C12 cells described above. Amido black staining (AB) of membranes was performed to confirm equivalent loading of chromatin between samples. **c** Representative immunofluorescence microscopy of C2C12 WT and shMMP-2 cells cultured in differentiation media for 4 days. A myosin heavy chain (MyHC) antibody was used to visualize myotube formation (red) and DAPI used to detect nuclei (blue). **d** Total number of MyHC positive cells (left) and the average number of nuclei/image (right) in the C2C12 WT and shMMP-2 cells were determined by analyzing 9 random fields from 3 independent biological replicates using a 20× objective. The percent of MyHC positive cells that were mononuclear or multinuclear in the C2C12 WT and shMMP-2 cells was also determined (bottom)

The results above predicted that persistent MMP-2 depletion would impair myogenic gene activation and, therefore, impair myoblast differentiation. To test this, a stable C2C12 cell line expressing an MMP-2-specific shRNA was generated [34]. The shMMP-2 and wild type (WT) control C2C12 cells were expanded to confluence before culturing in differentiation media for 4 days. Subsequent RT-qPCR analysis confirmed that the shMMP-2 cells displayed an ~ 80% reduction in MMP-2 expression relative to controls (Fig. 4a), strikingly similar to the siMMP-2 day 2 cells (Fig. 3a). Western analysis also confirmed that MMP-2 depletion in the shMMP-2 cells resulted in diminished H3NT proteolysis relative to controls (Fig. 4b). As predicted, persistent depletion of MMP-2-mediated H3NT proteolysis impeded activation of the canonical myogenic genes MyoG, MyoD, Mrf4 and Tnni2 (Fig. 4a).

To examine the phenotypic consequences of MMP-2 depletion on myoblast differentiation, immunofluorescence microscopy was performed in the day 4 C2C12 shMMP-2 and WT control cells. Cells were stained for the differentiation marker myosin heavy chain (MyHC) and counterstained with DAPI to visualize myotubes and nuclei, respectively (Fig. 4c). Consistent with impaired myogenic gene activation, the shMMP-2 cells displayed an ~ 50% reduction in the total number of MyHC+ cells compared to WT control, despite similar numbers of nuclei per field between the two samples (Fig. 4d). In contrast to the predominant long multinuclear myotubes observed in the WT control cells, the shMMP-2 MyHC + cells were typically shorter and ~ 75% were mononuclear (Fig. 4d). These results indicate that proficient myoblast differentiation is dependent on MMP-2.

### Nuclear MMP-2 H3NT protease activity facilitates myogenic gene activation

Although the data demonstrate that MMP-2 is required for H3NT proteolysis and myogenic gene activation, it remained unclear whether these were dependent on nuclear MMP-2 activity or MMP-2 activity in the ECM. If MMP-2 activity in the ECM is required, independent of nuclear MMP-2 activity, we hypothesized that restoring MMP-2 ECM activity in the shMMP-2 C2C12 cells would rescue H3NT proteolysis and myogenic gene activation. To test this, C2C12 shMMP-2 cells were expanded to confluence and subsequently cultured in MMP-2-rich conditioned media (CM) collected from differentiated wild type C2C12 cells (Fig. 3b, d). Total RNA and chromatin were collected 4 days after media replacement for analyses. Contrary to the hypothesis, RT-qPCR analysis demonstrated that supplementation of MMP-2 activity in the ECM failed to rescue H3NT proteolysis or myogenic gene activation (Fig. 4a, b). These results indicate that MMP-2 activity in the ECM is insufficient to induce myogenic gene activation and support the necessity of nuclear MMP-2 H3NT protease activity for myogenic gene activation.

## Discussion

Although increasing evidence supports H3NT proteolysis as a conserved epigenetic event in eukaryotic development, the H3NT proteases and biological significance of H3NT proteolysis remain largely unknown. The goal of this study was to identify and investigate an established developmental model system to gain new insights into the functional role of H3NT proteolysis. Here, we leveraged several cell line models of skeletal myogenesis where it was previously reported that, in contrast to the myoblast precursor cells, differentiated myotubes displayed a single H3cl product [11]. Consistent with these findings, our results demonstrate that H3NT proteolysis commences within 24 h following myogenic signaling and that the H3cl product progressively accumulates during differentiation of C2C12 and L6 myoblast cells. The timing of H3NT proteolysis was strikingly similar to an osteoclastogenesis cell model, where a single H3cl product was observed within 24 h following osteoclastogenic signaling of precursor cells and a progressive accumulation of the H3cl product was observed in differentiating osteoclasts [14]. Both myoblast and osteoclast precursor cells fuse to form large multinuclear myotubes and osteoclasts, respectively, suggesting that H3NT proteolysis is a consequence of cell fusion and multinucleation. However, the cell fusion-defective L6.G8 myoblast cells also displayed an H3cl product within 24 h after myogenic signaling that increased over time. These collective findings support the developmental signaling-dependent induction of H3NT proteolysis at earlier stages of the differentiation program.

To investigate the biological function of H3NT proteolysis during myoblast differentiation, we first sought to identify the H3NT protease responsible for these effects. We serendipitously discovered that the MMP-2 gelatinase is a novel H3NT protease and the principal H3NT protease of myoblast differentiation. Gelatin zymography demonstrated that MMP-2 activity is detected in nuclear extracts of differentiating C2C12 cells within 24 h following myogenic signaling and that MMP-2 activity progressively increased during differentiation, concomitant with the progressive increase of the chromatin-bound H3cl product. RNAi-mediated depletion of MMP-2 impaired H3NT proteolysis whereas rescue of MMP-2 activity restored H3NT proteolysis in differentiating myoblasts. These findings were strikingly similar to our previous report in an osteoclastogenesis cell model demonstrating that progressive increases in nuclear MMP-9 activity were concomitant with H3NT proteolysis during differentiation and depletion of MMP-9 impaired H3NT proteolysis [14]. Since the canonical functions of the gelatinases reside in the ECM, it was unexpected to discover that MMP-2 and MMP-9 function as nuclear H3NT proteases. However, nuclear gelatinase activity is not uncommon and has been reported in several different cell types including neurons, cardiac myocytes and liver cells [35]. These findings indicate that a fraction of newly synthesized MMP-2 or MMP-9 is transported to the nucleus, instead of the ECM, during myoblast differentiation and osteoclastogenesis, respectively. The mechanisms that facilitate their nuclear transportation remain unknown but, since both lack canonical nuclear localization signals, it is likely that unidentified nuclear chaperones are necessary for transport. Further studies are required to identify and investigate gelatinase-associated proteins that facilitate MMP-2 and MMP-9 nuclear localization. Regardless, our discovery that MMP-2 is the principal H3NT protease of myoblast differentiation provides additional evidence that distinct non-nuclear proteases are utilized as nuclear H3NT proteases in a differentiation-dependent context.

We previously reported that MMP-9-dependent H3NT proteolysis directly near the transcription start sites of canonical osteoclastogenic genes facilitates their activation and is necessary for proficient osteoclast differentiation [14]. Similarly, this study demonstrated that MMP-2-dependent H3NT proteolysis facilitates the activation of canonical myogenic genes necessary for proficient myoblast differentiation. These collective results suggest that distinct H3NT proteases are utilized in a developmental-dependent context to facilitate the activation of appropriate lineage-specifying genes necessary for proficient differentiation. Further studies are required to rigorously test the validity of this model. Since all currently known H3NT proteases have canonical functions outside the nucleus, it was unknown whether nuclear H3NT protease activity was essential or dispensable for proficient differentiation. The results of this study provide the first experimental evidence demonstrating that nuclear MMP-2 H3NT protease activity, independent of canonical MMP-2 activity in the ECM, is necessary for myogenic gene activation and myoblast differentiation. Further studies are required to determine if nuclear H3NT protease activity is sufficient to facilitate lineage-specifying gene activation and proficient differentiation.

## Conclusions

This study demonstrates that H3NT proteolysis is a conserved epigenetic program of skeletal myogenesis in vitro and in vivo. The data indicate that MMP-2 is a novel H3NT protease required for proficient myogenic gene activation and myoblast differentiation. The study also provides the first experimental evidence to support the necessity of nuclear H3NT protease activity in the activation of lineage-specifying genes.

## Materials and methods

### Cell culture

The mouse C2C12 (ATCC CRL-1772) and rat L6 (ATCC CRL-1458) myoblast cells were cultured as previously described [11]. Briefly, cells were passaged in DMEM (Corning 10-013-CV) supplemented with 10% FBS, 1 × GlutaMAX (ThermoFisher 35,050) and 1 × antibiotic–antimycotic (ThermoFisher 15,240). Cells were grown to confluence and induced to differentiate in DMEM without sodium pyruvate (Corning 10-017-CV) supplemented with 2% horse serum. Myotubes were isolated from the non-differentiated reserve cells using diluted trypsin. The cell permeable inhibitors E64 (25 μM, Selleckchem S7379), GM6001 (25 μM, Selleckchem S7157), MMP-9i (100 nM, Selleckchem S0769) and rapamycin (20 nM, Selleckchem, S1039) in DMSO were added to the media 1 day prior to and following media replacement.

### Light microscopy

The staining and visualization of myotubes was performed as previously described [36]. Briefly, cells were washed in PBS, fixed in 70% ethanol for 10 min and then stained with LADD (7.3 mg/ml toluidine blue 2.7 mg/ml fuchsin, 30% ethanol) for one minute. The cells were repeatedly washed in diH_2_O until the residual dye no longer leached into the water. The cells were dried and imaged using a KEYENCE BZ-X700. Images were acquired with a 10× objective and automatic white balance adjustment. Images obtained had a resolution of 1920 × 1440 pixels and were analyzed using Adobe Photoshop.

### Nuclear extraction and chromatin purification

Isolation of nuclear soluble extracts and purified chromatin was adapted from [37]. Cells were collected, washed in PBS and then incubated on ice in 1 ml Low-Salt Buffer (LSB) (25% glycerol, 20 mM HEPES pH 7.9, 1.5 mM MgCl_2_, 2 mM DTT, Halt Protease and Phosphatase Inhibitor Cocktail) for 15 min. Nuclei were isolated by adding the non-ionic detergent NP-40 (0.75% final) and gently passed through a 20 ga needle ten times. Nuclei were pelleted at 500 g for 2 min and then suspended in 500 µl LSB. The pelleted nuclear volume (PNV) was determined by measuring the total volume—500 µl LSB. Nuclei were pelleted and suspended in ½ PNV of LSB. A volume of ½ PNV of High-Salt Buffer (HSB) (25% glycol, 1.6 M NaCl, 20 mM HEPES pH 7.9, 1.5 mM MgCl_2_, 2 mM DTT, Halt Protease and Phosphatase Inhibitor Cocktail) was added dropwise while vortexing at low speed to achieve a final concentration of 400 mM NaCl. Samples were then incubated under agitation at 4 °C for 30 min and centrifuged at 21,000 g for 10 min. The supernatant was collected as the nuclear soluble extracts. The pellet (chromatin) was washed twice in the 400 mM NaCl buffer, pelleted and suspended in SDS-PAGE load dye (final: 50 mM Tris–HCl pH 6.8, 3% SDS, 10% glycerol, 5% β-mercaptoethanol, 0.002% bromophenol blue). The samples were heated to 90 °C for 10 min and cooled on ice three times before shearing the chromatin by sonication in preparation for SDS-PAGE. The nuclear soluble protein concentration was determined using a BCA Protein Assay Kit (ThermoFisher 23,225).

### Chromatin extraction from mouse muscle tissue

Chromatin was purified from adult C57BI6 mouse skeletal muscle as previously described [28] (IACUC #20,633). Bulk muscle from the hind limb thigh was placed on a glass plate with 4 ml of buffer A (0.25 M sucrose, 10 mg/ml BSA, 5 mM MgCl_2_, Halt Protease and Phosphatase Inhibitor Cocktail) and minced into small pieces (< 1 mm) using a razor. Tissue was placed in a dounce homogenizer with 5 ml of buffer A and ground for 50 strokes with a loose pestle. The homogenate was passed through a 100-micron filter to remove bulk cellular debris, placed in a clean dounce homogenizer with Triton X-100 (0.5% final) and further ground for 50 strokes with a tight pestle. The homogenate was passed through a 40-micron filter and centrifuged at 500 g for 10 min. To isolate nuclei from the remaining cellular debris, the pellet was suspended in 3.3 ml of Buffer C (10 mM NaCl, 3 mM MgCl_2_, 10 mM Tris–HCl, pH 7.5, 1% NP-40) and layered over a sucrose cushion (1.8 M Sucrose, 3 mM Mg(Ac)_2_, 10 mM Tris–HCl, Halt Protease and Phosphatase Inhibitor Cocktail, pH 8.0) in microfuge tubes. The tubes were centrifuged at 2500 g for 10 min and the nuclei-rich pellet was suspended in LSB. Chromatin was isolated as described above.

### Western analysis

Chromatin samples were fractionated on a 4–14% SDS-PAGE gel, transferred to nitrocellulose membrane using Towbin buffer (25 mM Tris pH 8.8, 192 mM Glycine, 20% methanol) on a Hoefer TE 77 semi-dry transfer unit for 2 h at 55 mA/membrane. The membrane was incubated in blocking solution (5% non-fat milk in TBS) for 1 h prior to replacement with primary antibody diluted in TBS with 1.5% non-fat milk: anti-Histone H3 (abcam ab1791, 1:10,000), anti-Histone H2B (abcam ab1790, 1:1000), anti-Histone H2A (abcam ab18255, 1:1000), anti-Histone H4 (abcam ab10158, 1:1000), anti-Histone H3K18ac (Active Motif 39,755, 0.5 μg/ml) or anti-Histone H3K27me2 (Active Motif 39,245, 1:1000). Membrane was incubated on a rocker overnight at 4 °C, washed in TBS-T (TBS, 0.1% Tween-20) three times for 10 min and incubated in TBS with 1.5% non-fat milk containing a secondary goat anti-rabbit IgG Alexa Fluor conjugated antibody (Life Technologies A27042, 1:15,000) for 45 min at room temperature. The membrane was washed in TBS-T three times for 10 min, rinsed in diH_2_O and visualized using a LI-COR Odyssey. Membrane was then stained with 0.1% amido black 10B (Sigma N3393) in 10% acetic acid for 1 min, destained with 5% acetic acid twice for 1 min and rinsed in diH_2_0 twice for 10 min before imaging using a LI-COR Odyssey.

### In vitro* H3 cleavage assay*

Nuclear soluble extracts (1 μg) were incubated with acid extracted core histones purified from C2C12 myoblasts (1 μg) in Cleavage Buffer (50 mM Tris–HCl pH 7.5, 20 mM CaCl_2_, 10 mM KCl, 5% glycerol) at 37 °C for 2 h. Protease inhibitors were incubated with nuclear soluble extracts for 15 min at room temperature before adding the histone substrates: aprotinin (1 ng/ml), leupeptin (5 ng/ml), pepstatin (0.5 ng/ml), E64 (10 nM) or EDTA (25 mM). SDS-PAGE and Western analysis was performed as described above.

### Gelatin zymography

A 4–10% SDS-PAGE gel was cast with 0.1% gelatin in the resolving portion of the gel (10% polyacrylamide, 1 mg/ml gelatin, 375 mM Tris–HCl pH 8.8, 0.1% SDS). The gel lanes were loaded with equivalent amounts of nuclear soluble extracts in 1 × non-denaturing load dye (66 mM Tris–HCl pH 6.8, 10% glycerol, 2% SDS, 0.01% bromophenol blue) and fractionated via electrophoresis. The gel was incubated in 100 ml renaturing solution (50 mM Tris–HCl pH 7.5, 5 mM CaCl_2_, 2.5% Triton X-100) two times for 30 min to remove SDS, washed in 100 ml diH_2_O two times for 15 min, and incubated in 100 ml developing solution (50 mM Tris pH 7.5, 10 mM CaCl_2_, 0.02% NaN_3_) for 1 h. The developing solution was replaced and the gel was incubated at 37 °C for 18 h. The gel was incubated with 100 ml of staining solution (5% methanol, 10% acetic acid, 0.125% coomassie R-250) for 4 h and then 100 ml of destaining solution (10% MeOH, 5% acetic acid) prior to imaging using a LI-COR Odyssey.

### RNAi

The MMP-2 siRNA (Qiagen, Mm_MMP2_3, SI01314033) and Non-Specific siRNA control (Qiagen, AllStars Neg. Control, SI03650318) were reverse transfected in 24-well plates using 10 pmol of siRNA and Lipofectamine RNAiMAX diluted in Opti-MEM, according to the manufacturer’s instructions (Life Technologies 13,778). Following complex formation, 2 × 10^5^ C2C12 cells were seeded in each well and incubated for 2 days to reach confluence prior to differentiation. One million C2C12 cells were electroporated with 20 μg of MMP-2 shRNA plasmid (Sigma TRCN0000031228) using the Cell Line Nucleofector Kit V and Nucleofector 2b device according to the manufacturer’s instructions (Lonza VVCA-1003). Cells containing the MMP-2 shRNA plasmid were selected with 2 μg/ml puromycin for at least 5 days prior to experimentation.

### RNA isolation and RT-qPCR

Total RNA was extracted from cells using TRIzol reagent according to the manufacturer’s instructions (ThermoFisher 15,596). Briefly, cells were washed in PBS before adding TRIzol directly to the cells. Cells were scraped and the mixture transferred to a microfuge tube. Chloroform (5:1 v/v) was added, the samples were vortexed, centrifuged and the RNA-containing aqueous phase was isolated. Isopropanol precipitation of the aqueous phase was performed at room temperature. RNA was pelleted by centrifugation and washed with 70% ethanol. The dried pellet was suspended in nuclease free diH_2_0 and the RNA concentration was determined by spectrophotometry. One microgram of total RNA was reversed transcribed using the SuperScript IV VILO Master Mix according to the manufacturer’s instructions (ThermoFisher 11,756). The qPCR was performed in triplicate using 10 ng of the resulting cDNA, PerfeCTa SYBR Green FastMix (Quantabio 95,072) and 5 μM of each of the following primers: MMP-2 (F: 5′-ACCCTGGGAGAAGGACAAGT, R: 5′-ATCACTGCGACCAGTGTCTG), MyoG (F: 5′-CCGAGCCTCTACAACAGGAG, R: 5′-GGTAACTTCCTCACCCACGA), MyoD (F: 5′-GGCTCTCTCTGCTCCTTTGA, R: 5′-AGTAGGGAAGTGTGCGTGCT), Mrf4 (F: 5′-GGCTGGATCAGCAAGAGAAG, R: 5′-AAGAAAGGCGCTGAAGACTG), Tnni2 (F: 5′-GAGATGAGGAGAAGCGCAAC, R: 5′-CGCTATCTGGAGCATCACAC), 18S (F: 5′-CTCTGTTCCGCCTAGTCCTG, R:5′-AATGAGCCATTCGCAGTTTC). Quality and specificity of the primer sets were validated by melt curve analysis. Expression of each gene was calculated using 2^(−ΔΔCt) relative to the 18S housekeeping gene, which remains invariant during myoblast differentiation. Three independent biological replicates were performed to determine the average expression change and standard deviation.

## Immunofluorescence microscopy

C2C12 cells were seeded in 4-well chamber slides (Nunc Lab-Tek II CC^2^) at a density of 5 × 10^4^ per well for 2 days to reach confluency. Four days following media replacement, cells were washed twice with PBS and fixed in 4% paraformaldehyde for 10 min. Cells were washed 3 times for 10 min in PBS and permeabilized with 1% Triton X-100 in PBS (v/v) for 5 min. Following 3 washes with PBS, slides were incubated for 1 h in blocking solution: PBS with 0.1% Triton X-100 and 3% BSA. Slides were then incubated in blocking solution with a mouse monoclonal MyHC antibody (MF20, 1:200, DSHB) for 2 h at room temperature and then washed with blocking solution 3 times for 10 min. Slides were incubated in blocking solution with a goat anti-mouse Alexa Fluor 568 secondary antibody (8 μg/ml, ThermoFisher A-11031) in blocking solution for 1 h in the dark, washed with PBS 3 times for 10 min, rinsed with diH_2_0 and dried. Slides were mounted on coverslips using VECTASHIELD PLUS with DAPI (Vector Laboratories H-2000) and visualized on a Zeiss Imager Z1 fluorescent microscope using a 20× objective. Three images from each of three independent biological replicates were analyzed to determine the average number of nuclei/image (DAPI) and the total number of MyHC + cells in all 9 images.

## Data Availability

All key data supporting the findings of this study are available in the paper. Additional data and materials are available from the corresponding author upon request.

## Abbreviations

H3NT: Histone H3 N-terminal tail
H3cl: Histone H3-cleaved product
MMP: Matrix metalloproteinase
ECM: Extracellular matrix
RNAi: RNA interference
PTM: Post-translational modification
mESC: Mouse embryonic stem
RANKL: Receptor activator of NF-kB ligand
MB: Myoblasts
MT: Myotubes
SM: Skeletal muscle
siRNA: Small interfering RNA
shRNA: Short hairpin RNA
RT-qPCR: Reverse transcriptase, quantitative polymerase chain reaction
WT: Wild type
MyHC: Myosin heavy chain
CM: Conditioned media
